# Supplementary material for: Recurrent neural networks with explicit representation of dynamic latent variables can mimic behavioral patterns in a physical inference task
Source: Nat Commun. 2022 Oct 4;13:5865. doi: 10.1038/s41467-022-33581-6 (PMC9532407; doi:10.1038/s41467-022-33581-6)
Supplement: Supplementary file 1 — Supplementary Information [file 41467_2022_33581_MOESM1_ESM.pdf]

Recurrent neural networks with explicit representation of dynamic latent variables can mimic behavioral patterns in a physical inference task

[1]Rishi Rajalingham, [1,2]Aida Piccato, and [1,2]Mehrdad Jazayeri

[1]McGovern Institute for Brain Research, [2]Department of Brain & Cognitive Sciences,

Massachusetts Institute of Technology, Cambridge, Massachusetts 02139, USA

Correspondence: mjaz@mit.edu

Supplemental Information

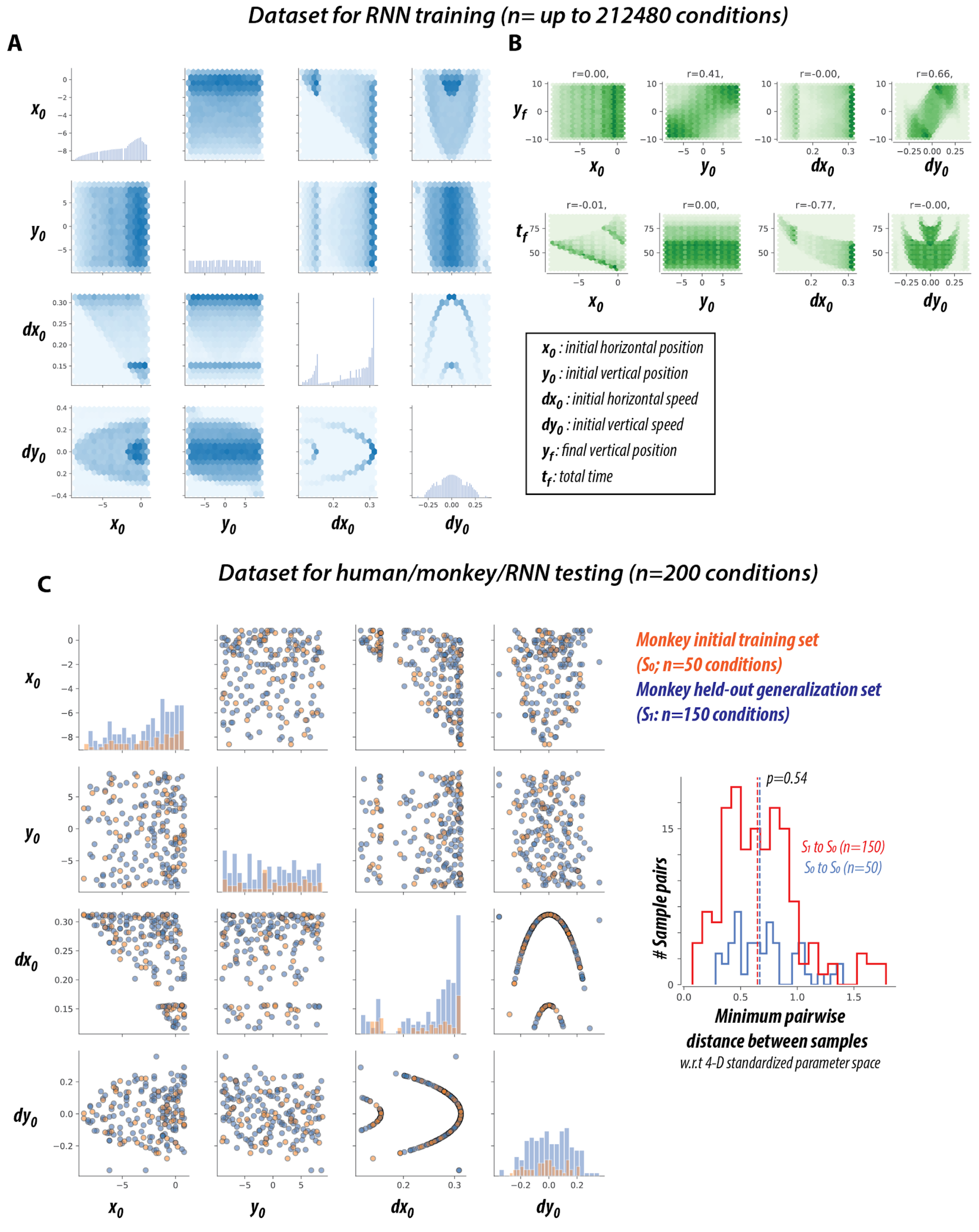

Figure S1. Datasets. (A) Distribution of meta-parameters corresponding to initial position (x_0,y_0) and initial velocity (dx_0, dy_0) for the task RNN training set. The diagonal panels correspond to histograms of each meta-parameter.

The off-diagonal panels correspond to 2D histograms showing the joint distribution for each pair of meta-parameters.

(B) Each panel shows the joint distribution of each meta-parameter with the target output (final ball position, y_f, see top panels) and the time to interception (t_f, see bottom panels).

(C) (left) Distribution of the same meta-parameters for the test set of 200 unique conditions used to characterize human, monkey, and RNN behavior, with formatting similar to (A). The meta-parameter distributions are separated by color by further randomly split into two sets with regards to monkey behavior: a small set of 50 conditions used to first train monkeys (here termed S_0), and a held-out generalization set of 150 conditions for testing first-trial generalization (here termed S_1). (right) To examine how "overlapping" the two sets S_0 and S_1 are, we measured the minimum pairwise distance between conditions within and across these two sets, and compared the magnitude of these distances. This is akin to asking: how similar is a new sample to the closest training sample? If the generalization set required a "strong extrapolation," this would result in minimum pairwise distances across tests that are much larger than the pairwise distances within sets. In contrast, if the generalization set only required trivial "memorization" strategies, this would result in minimum pairwise distances across tests that are much smaller than the pairwise distances within sets. Importantly, it is not clear how to estimate such distances, i.e. with respect to what representations (e.g. the time-course of pixel inputs, the time-course of latent variables, the initial.state, etc.). Here, we picked one such representation, the 4D vector of ball meta-parameters (x_0,y_0,dx_0, dy_0) for each condition, and standardized each meta-parameter to ensure that all four contributed equally; Euclidean distances on this standardized representation correspond to Mahalanobis distances over the raw 4D representation. With respect to this choice, the histograms show that the pairwise distances within the training set are not statistically different to the pairwise distances across training and test sets (blue vs red, $p=0.54$, two-tailed two-sample t-test). This result suggests that, at least with respect to this characterization, the held-out conditions were approximately as different to the previously trained conditions, as those training conditions were to each other.

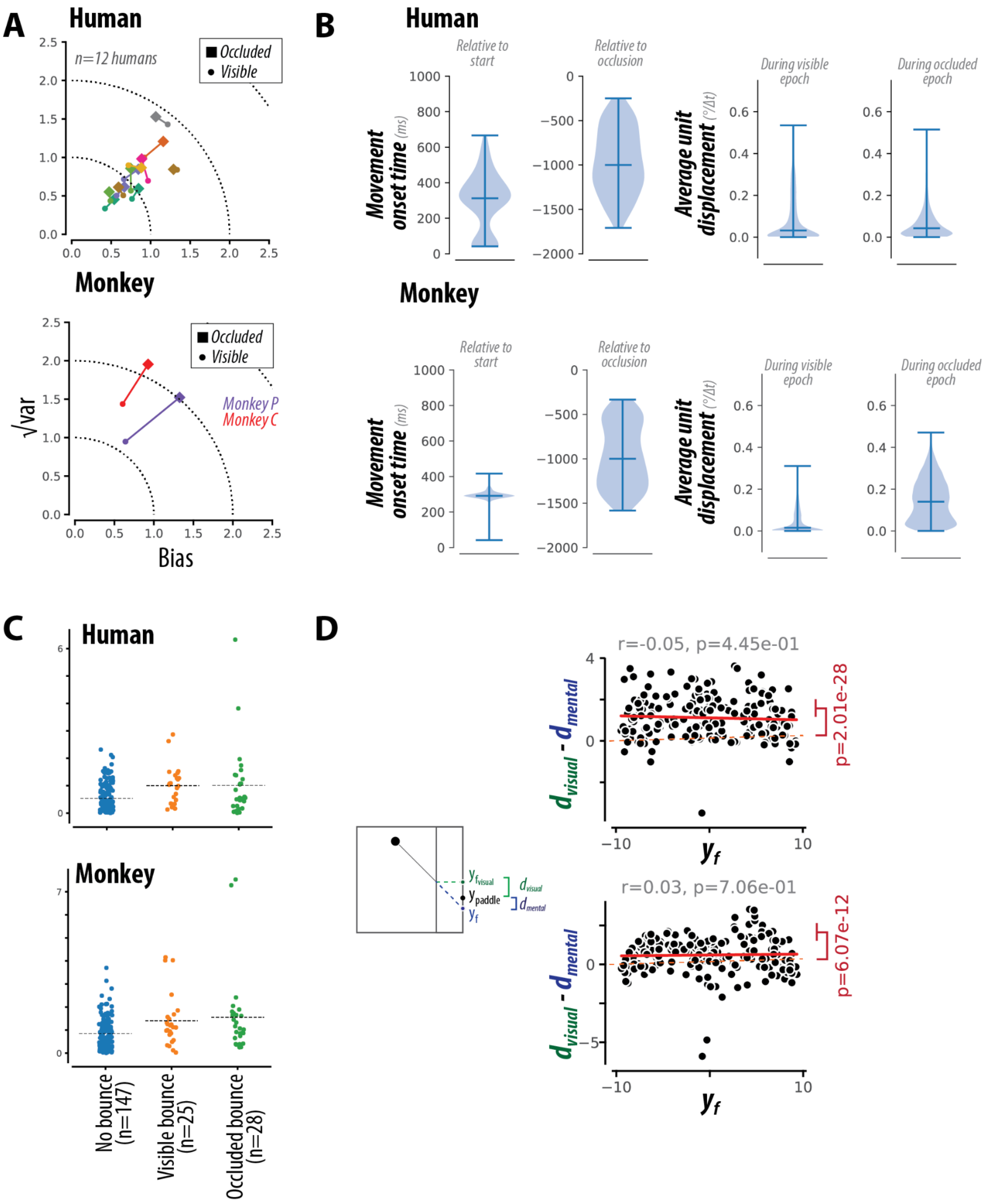

Figure S2. Behavioral performance. (A) Bias (absolute error, after averaging across trials of the same condition) and variance (variability across trials of the same condition) for individual humans (top) and monkeys (bottom), shown separately for visible (circle) and occluded (square) trials. The dotted circles correspond to lines of equal root-mean-squared error (RMSE). As expected, errors are lower on visible trials.

(B) The two left columns show the distribution of movement onset times relative to the beginning of the trial (left) and to the beginning of the occluded epoch (right) for humans (top) and monkeys (bottom). The two right panels show the distribution of unit displacement during the visible (left) and occluded (right) epochs. Unit displacement was estimated from the instantaneous paddle position by first averaging across trials of the same conditions, and then measuring the mean absolute change in position.
(C) Dependence of error patterns for trials with no bounce, trials with a visible bounce, and trials with occluded bounce for humans (top) and monkeys (bottom). Given the relatively small number of conditions with visible and occluded bounces (25 and 28, respectively), it is difficult to estimate the specific effect of bounces. However, we observe that bounces result in larger errors, and conditions with occluded bounces have a larger range of errors.
(D) Error patterns could not be explained by an alternative visual tracking strategy, wherein the final paddle position is estimated based on the last visible position of the ball ($y_{f\_visible}$). To demonstrate this, we compared the distance between the average final paddle position to each of the candidate final positions, as predicted by visual tracking (d_{visual}) and mental tracking (d_{mental}) strategies. We observe that the corresponding bias (d_{visual} - d_{mental}) is significantly greater than zero (see red annotation, two-tailed t-test) and moreover is not dependent on the final ball position (as might be expected if this bias is somehow driven by boundary conditions, see gray title annotation), demonstrating that behavioral error patterns are inconsistent with a simpler visual tracking strategy.

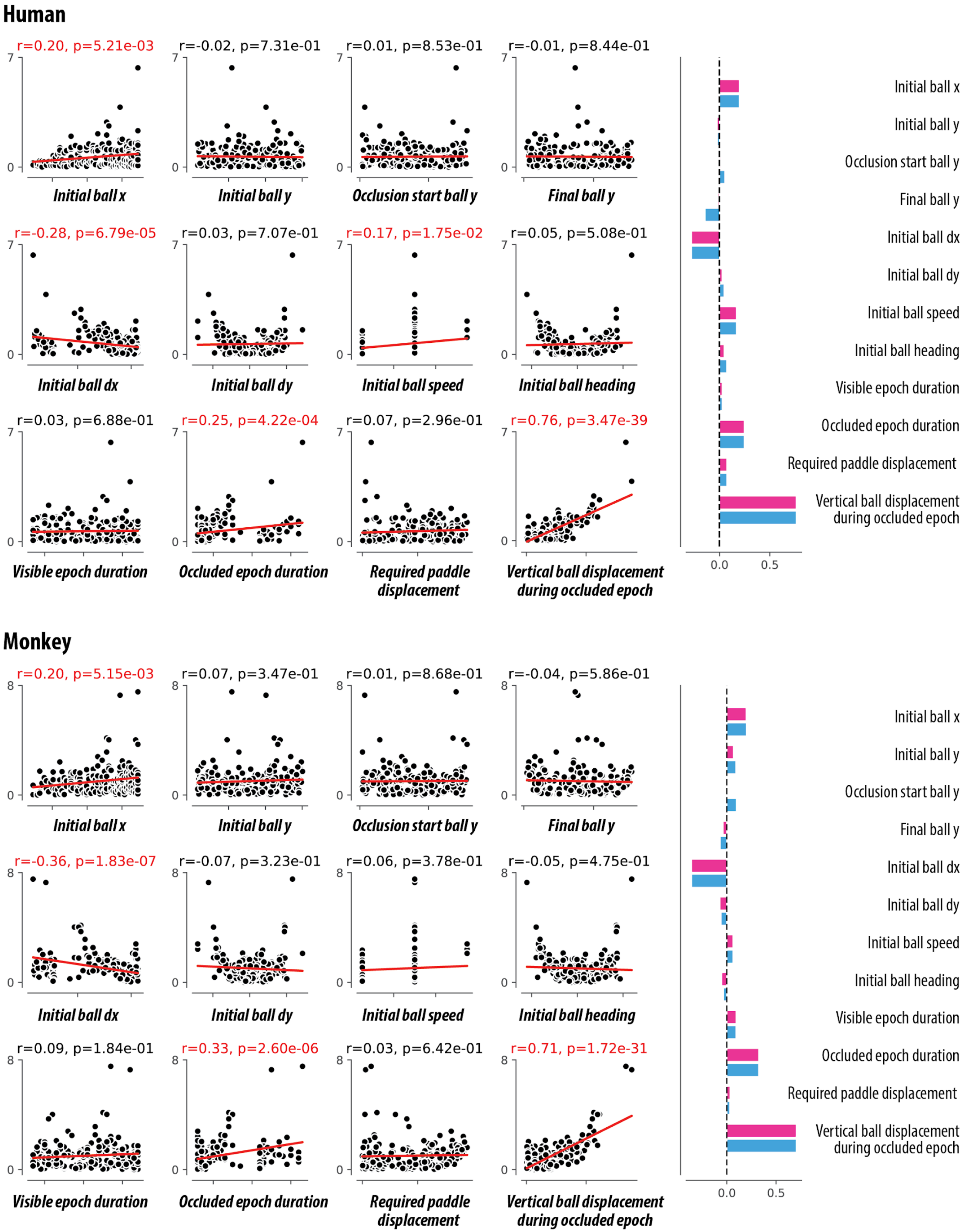
Human
r=0.20, p=5.21e-03
r=-0.02, p=7.31e-01
r=0.01, p=8.53e-01
r=-0.01, p=8.44e-01
Initial ball x
Initial ball y
Occlusion start ball y
Final ball y
r=-0.28, p=6.79e-05
r=0.03, p=7.07e-01
r=0.17, p=1.75e-02
r=0.05, p=5.08e-01
Initial ball dx
Initial ball dy
Initial ball speed
Initial ball heading
r=0.03, p=6.88e-01
r=0.25, p=4.22e-04
r=0.07, p=2.96e-01
r=0.76, p=3.47e-39
Visible epoch duration
Occluded epoch duration
Required paddle displacement
Vertical ball displacement during occluded epoch
Initial ball x
Initial ball y
Occlusion start ball y
Final ball y
Initial ball dx
Initial ball dy
Initial ball speed
Initial ball heading
Visible epoch duration
Occluded epoch duration
Required paddle displacement
Vertical ball displacement during occluded epoch
0.0
0.5
Monkey
r=0.20, p=5.15e-03
r=0.07, p=3.47e-01
r=0.01, p=8.68e-01
r=-0.04, p=5.86e-01
Initial ball x
Initial ball y
Occlusion start ball y
Final ball y
r=-0.36, p=1.83e-07
r=-0.07, p=3.23e-01
r=0.06, p=3.78e-01
r=-0.05, p=4.75e-01
Initial ball dx
Initial ball dy
Initial ball speed
Initial ball heading
r=0.09, p=1.84e-01
r=0.33, p=2.60e-06
r=0.03, p=6.42e-01
r=0.71, p=1.72e-31
Visible epoch duration
Occluded epoch duration
Required paddle displacement
Vertical ball displacement during occluded epoch
Initial ball x
Initial ball y
Occlusion start ball y
Final ball y
Initial ball dx
Initial ball dy
Initial ball speed
Initial ball heading
Visible epoch duration
Occluded epoch duration
Required paddle displacement
Vertical ball displacement during occluded epoch
0.0
0.5

Figure S3. Behavioral performance. (right) Each panel shows absolute average endpoint error against task parameters varying per condition. The strength of each dependence, measured via a Pearson correlation, is shown on the corresponding panel titles; significant dependences are highlighted in red. (left) The raw Pearson correlation and the partial Pearson correlation, after conditioning on the final ball position, are shown for each task parameter on the bar plot. If errors were a result of a simple motor cost, we would predict that trials that require large movements (where the final ball position is far from the initial paddle position) would have larger errors on conditions; this is in fact not true (see "required paddle displacement" panel). If errors were a result of movement preparation, we would predict that trials with longer visible epochs (and thus longer preparatory time) would have smaller errors on average; this is in fact not true (see "visible epoch duration" panel). If errors were a result of visuo-spatial biases, we would predict that errors would depend on the initial, intermediate, or final vertical position of the ball; this is in fact not true (see "initial ball y", "occlusion start ball y" and "final ball y" panels). Instead, error patterns depended largely on dynamic variables relating to the ball speed (see "initial ball dx", "initial ball speed", and "occluded epoch duration" panels); we additionally observe a non-monotonic relationship with vertical ball velocity (see "initial ball d_y" panel). Given the correlation between initial x position and ball speed built into this dataset (see Figure S1), errors were additionally correlated to the initial x position (x_0). These dependencies are consistent with a noisy simulation model wherein errors in dynamic inference accumulated on a moment-by-moment basis during the occluded epoch. Notably, if errors were a result of such error accumulation, we would predict that trials where the vertical ball displacement during the occluded epoch is larger would have larger errors on average; we found this to be true (see "vertical ball displacement during the occluded epoch" panel).

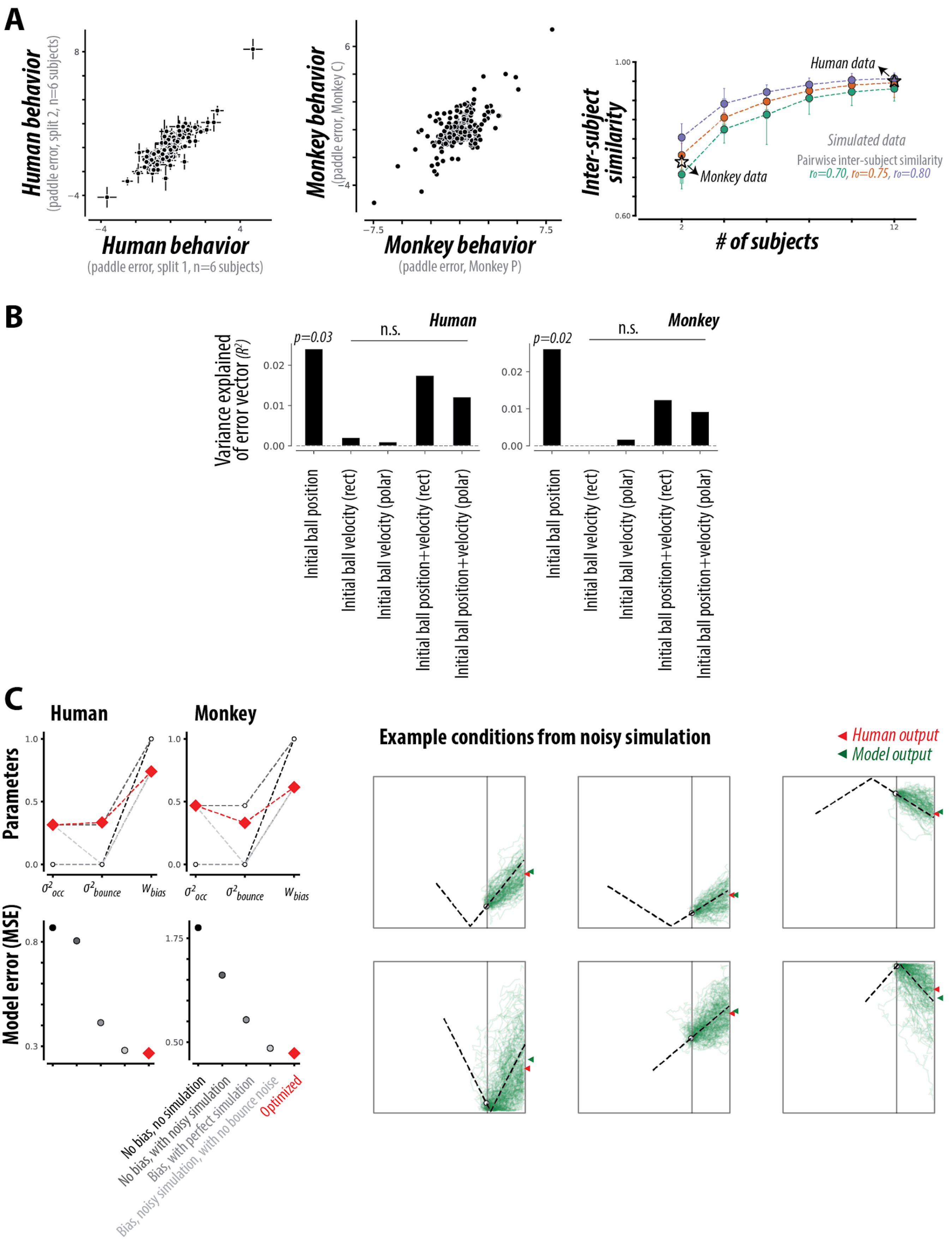

Figure S4. Behavioral error patterns. (A) Comparison of error across spit-halves of data for the 12 human participants (left) and the 2 monkeys (center). (right) Sample size effect. We ran simulations to characterize the

correlations between split halves as a function of sample size. We generated 200-dimensional vectors (to match the error vector dimensions) with pairwise correlations of approximately r_0. We then measured our similarity measure for disjoint split halves, averaged the values, and measured the correlation between these two vectors to estimate the effect of sample size. The dashed curves show the average similarity estimates for different r_0. This analysis is consistent with a correlation of ~0.75 for both monkeys and humans.

(B) Error patterns could not be explained by a simple function of initial position and velocity. We used cross-validated linear regression to predict the error pattern from the initial ball position and ball velocity (top), and the resulting prediction was not better than expected by chance ($R2 < 0.05$ for all, $p>0.05$ for all except initial position).

(C) To characterize primate behavior, we constructed process models that explicitly "simulated" the moment-by-moment ball position during the occluded epoch in the presence of noise, and fit the model parameters to human and monkey behavior. We found that the optimal parameters corresponded to noisy simulations with a moderate bias towards the last visible ball position (see red diamonds, top left panels). To contextualize this optimized model, we simulated specific control models that incorporated some of the key characteristics (e.g. "No bias, no simulation", "No bias, with noisy simulation", etc.; see gray circles, top left panels). As expected (by definition), the optimized model fits the primate data better than all of these controls (see bottom left panels). This analysis reveals that both noisy simulation and a bias towards the last visible ball position are necessary to capture primate behavior. The panels on the right show six example conditions, directly comparing the noisy process models with human behavior. Each panel shows the M-Pong frame, with the occluder delineated in gray, and the ground truth ball position during the occluded epoch shown with a dashed black line. Each green trace shows a single simulation run through the process model. The green arrow shows the model's output, obtained by averaging the endpoint locations of all simulation runs. The red arrow shows the average human endpoint paddle position.

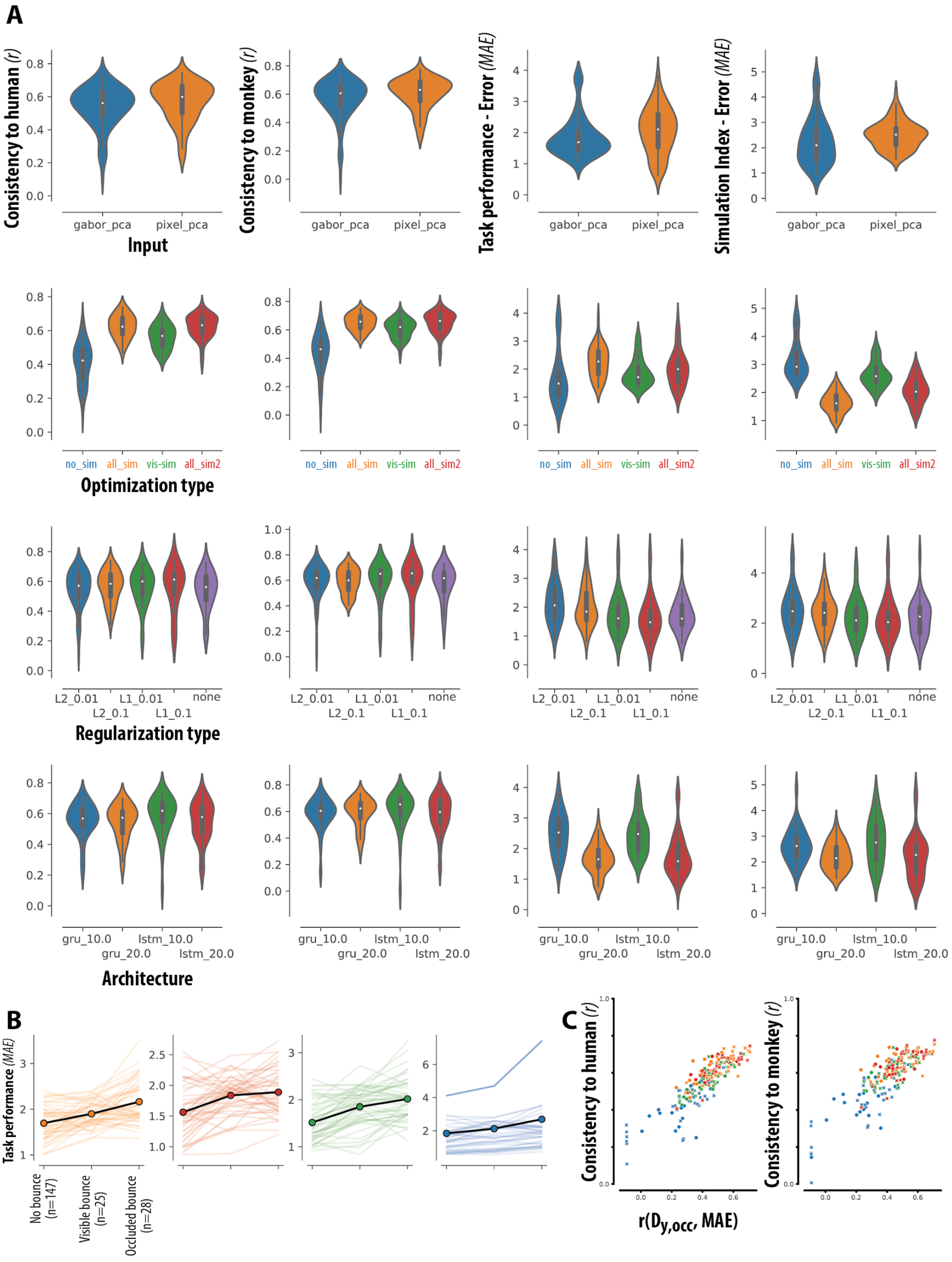
A
Consistency to human (r)
Consistency to monkey (r)
Task performance - Error (MAE)
Simulation Index - Error (MAE)
gabor_pca
pixel_pca
Input
no_sim
all_sim
vis-sim
all_sim2
Optimization type
L2_0.01
L2_0.1
L1_0.01
L1_0.1
none
Regularization type
gru_10.0
gru_20.0
lstm_10.0
lstm_20.0
Architecture
B
Task performance (MAE)
No bounce (n=147)
Visible bounce (n=25)
Occluded bounce (n=28)
C
Consistency to human (r)
Consistency to monkey (r)
r(Dy,occ, MAE)

Figure S5. RNN hyper-parameters. (A) Different RNN models varied with respect to several hyper-parameters: different cell types (***rnn_type****: LSTM or GRU*), number of cells (***n_hidden****: 10 or 20*), input representation types (***input****: pixel_pca or gabor_pca*), and regularization types (***reg****: L1_0.01, L1_0.1, L2_0.01, L2_0.1, or none*); and were differently optimized (***loss_weight_type****: no_sim, vis_sim, all_sim, or all_sim2*). The sixteen panels show the effect of each of the hyperparameter choices on task performance, simulation index, and primate consistency (with respect to both human and monkey behavior). The precise number of models per distribution varies for each of the sixteen panels, which group n=232 models in total along different dimensions. Each distribution is shown via a colored violin-plot (obtained by a kernel density estimate) as well as a gray boxplot. The range, maxima, and minima of the data are shown by the boxplot whiskers. The 25th, 50th, and 75th percentiles of the distribution are shown by the boxplot box. **(B)** Dependence of performance on bounces, separated by visible and occluded bounces (i.e. bounces that occurred during the visible and occluded epochs, respectively). Each panel shows this dependence for all tested RNN models of each of the four optimization types, with colors as in Fig 3C; thin lines correspond to individual RNN models, and dark lines correspond to the mean over all RNNs of a given optimization type. **(C)** In Figure S3C, we found that conditions where the vertical ball displacement during the occluded epoch is larger tended to yield larger errors for both humans and monkeys, consistent with a biased noisy simulation. Here, we tested whether this was true for RNNs, and estimated the correlation between their error (MAE) and the vertical ball displacement during the occluded epoch ($D_{y,occ}$) for each tested RNN model. The scatter shows that this estimated correlation is highly predictive of the consistency scores of RNNs, with respect to both human (left) and monkey (right) behavior. In other words, the similarity in error patterns between RNNs and primates is largely explained by this common feature.

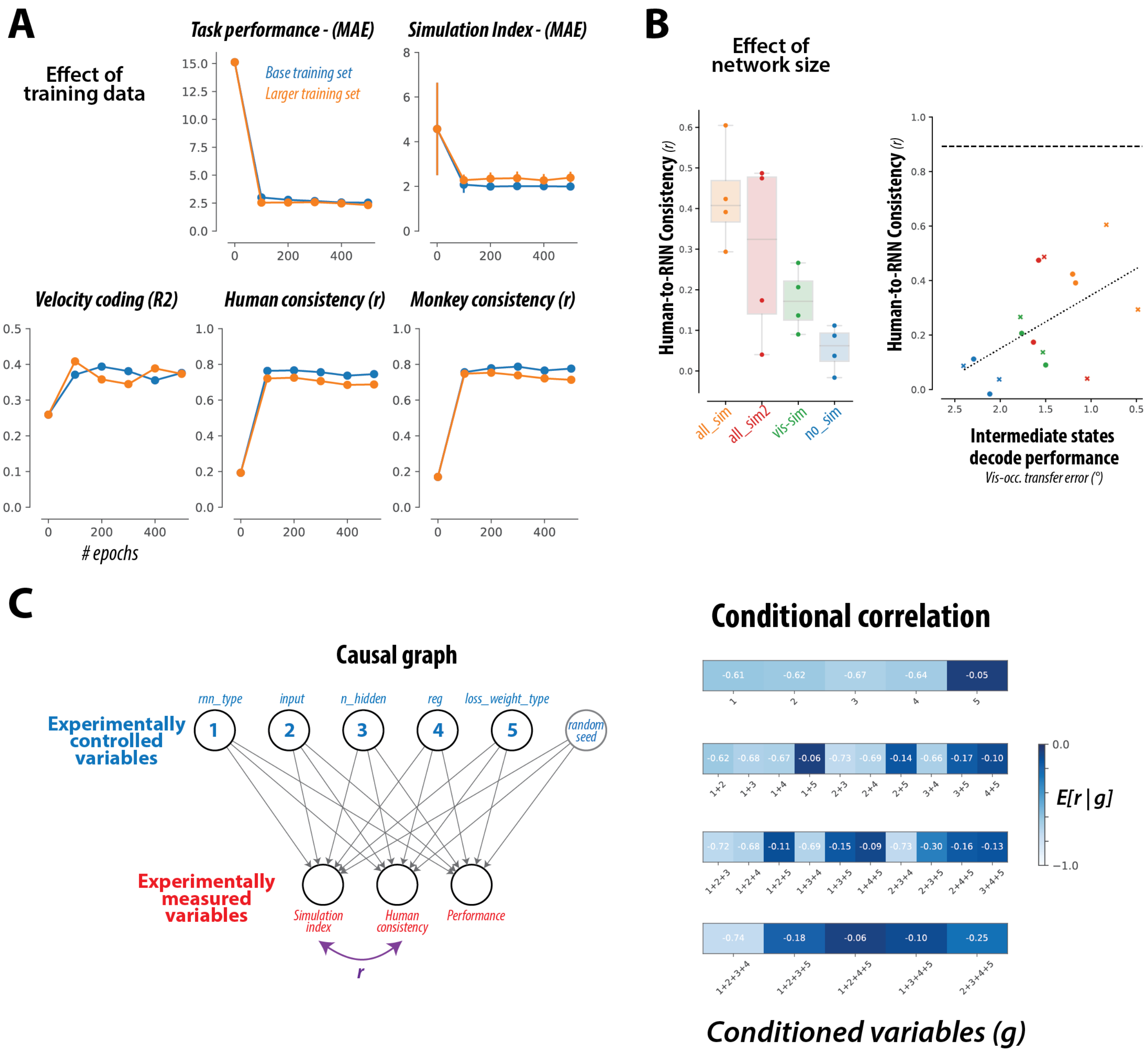

Figure S6 RNN controls. (A) Effect of RNN optimization. For the RNN model architecture with the highest human-consistency score, we evaluated key RNN metrics (e.g. performance, simulation index, consistency to humans and to monkeys) while varying both the number of training epochs and the training data (number of training samples and distribution of training data). We found that these metrics were largely insensitive to such variations in RNN optimization, suggesting that the extent of RNN training was sufficient to converge upon "stable" network solutions, and that our key results and inferences are largely robust of the details of this optimization procedure. Each point corresponds to the metric of a single (n=1) model estimated over n=200 Mental-Pong conditions. Error bars correspond to mean ± SE, estimated via bootstrapping over conditions.

(B) Effect of RNN size. Starting with the RNN model architecture with the highest human-consistency score, we varied its architecture by testing networks with 100 units and 200 units of both LSTM and GRU types. These networks exhibited qualitatively similar results, whereby networks optimized for dynamic inference ability were most primate-like in their behaviors (left panel), and human consistency scores were correlated to dynamic inference

ability (right panel). The left panel shows the distribution of human consistency scores for each network optimization type; the range, maxima, and minima of the data are shown by the boxplot whiskers, and the 25th, 50th, and 75th percentiles of the distribution are shown by the box.

(C) The left panel shows the causal graph of our RNN experiments. We experimentally controlled five different RNN hyper-parameters (top row, blue), and from the resulting RNN model instances, we measured several attributes (bottom row, red), including the simulation index and the human consistency. To uncover which RNN hyper-parameters *cause* the strong negative correlation (r) between simulation index and human consistency, we measured the conditional correlation E[r|g], conditioning on each of the 30 possible combinations (g) of the five hyper-parameter types. The right panel shows the resulting conditional correlations for each of the 30 combinations. Darker values correspond to smaller magnitude correlations. The observed correlation between the simulation index and human consistency is largely driven by the hyper-parameter defining the optimization target (***loss_weight_type***).

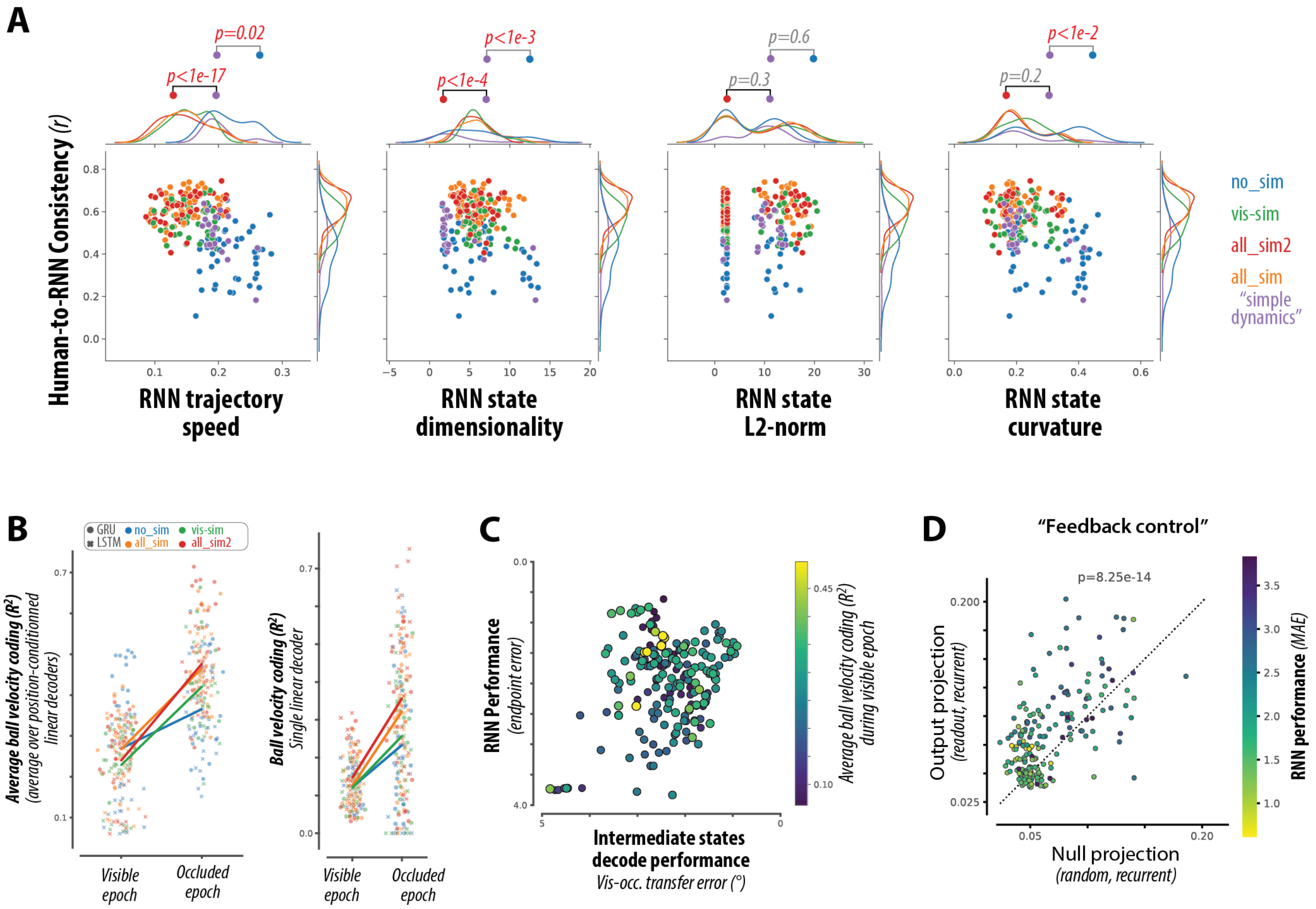

Figure S7 RNN dynamics. (A) As a control, we optimized a new set of RNN models on task performance with additional regularization to promote simple dynamics, by adding regularization terms related to the L2-norms of the hidden state activity and the derivative of the hidden state activity (see Methods). To verify that this regularization had the intended effect on RNN representations, the four panels show the distribution of human-consistency against four representational metrics (dimensionality, speed, curvature, and norm) for all trained RNN models, grouped by their optimization type. Statistical comparisons between relevant distributions are shown above each scatter (unpaired t-test). The top comparison corresponds to 'no_sim' vs 'simple_dynamics' (blue vs purple), and the bottom comparison corresponds to 'all_sim2' vs 'simple_dynamics' (red vs purple). We observe that for all metrics that differed across RNN types, the 'simple_dynamics' RNNs (purple) did indeed diverge from the baseline 'no_sim' (blue) models as intended.

(B) Comparison of average velocity coding during visible and occluded epochs, for all RNN models. The left panel shows velocity coding estimated via position-conditioned linear decoders, whereas the right panel shows the corresponding estimates with a single position-independent linear decoder.

(C) Average velocity coding during visible epoch is not correlated to either performance or simulation index, in contrast to the corresponding metric during the occluded epoch (see Figure 5E).

(D) We estimated a measure of "feedback control" to characterize the alignment between the read-out weights and the recurrent weights. While RNNs do not receive explicit instantaneous visual feedback, this metric aims to capture the extent to which the output of the network is fed back into its activity. The scatter plot shows the comparison of

this metric against the null, for each RNN model; marker color corresponds to model performance. We observed that the median amount of feedback control was significantly greater than expected by chance.

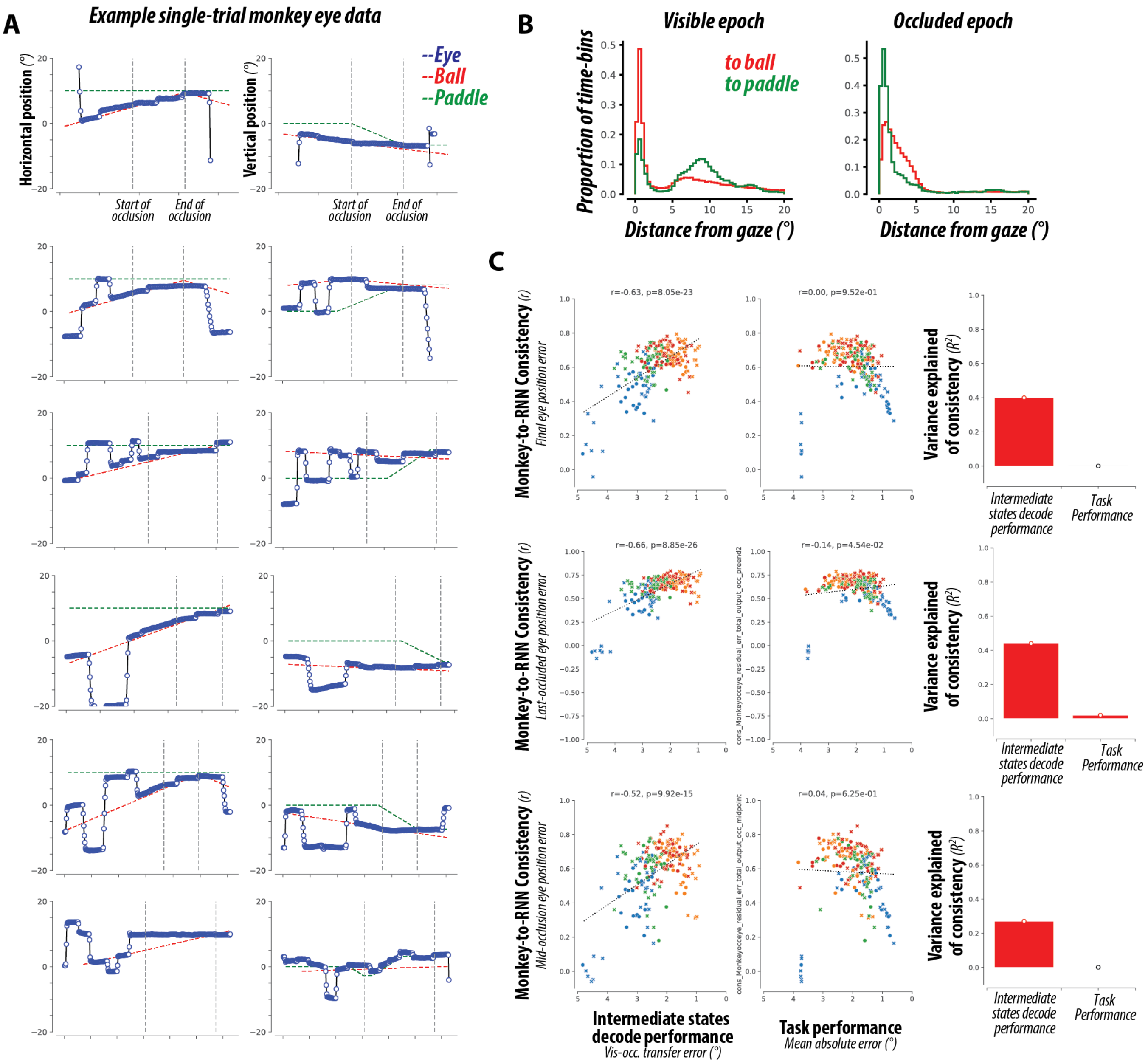

Figure S8. Eye data. (A) Example single-trial eye traces for six different trials of six different occluded M-Pong conditions. Note that we focus on monkey data for eye movement analysis where we had higher-fidelity eye tracking measurements (due to head-fixation and repeatedly calibrated). The left and right panels correspond to horizontal and vertical position, respectively. Each panel shows the time-course starting from the beginning of the trial; the vertical dashed lines correspond to the start and end of the occluded epoch. The eye trace is shown in blue, while the ball and paddle are shown in red and green respectively. We qualitatively observe sporadic saccadic eye movements that place the center of gaze on either the ball or the paddle, or occasionally on neither. We note that tracking the occluded ball via smooth pursuit eye movements is virtually impossible (as pursuit movements can only be made to visible targets, except in very rare experimental conditions).

(B) Each histogram shows the frequency of eye-to-ball and eye-to-paddle distances during the visible and occluded epochs. During the visible epoch, monkeys typically either fixated on the center or tracked the visible ball (left panel, red) and sparingly tracked the paddle. During the occluded epoch, they typically either fixated on the center or tracked the moving paddle (left panel, green) and rarely made eye movements to the location of the invisible ball.

(C) We repeated the primary analyses of Figure 3 (F,G,H) using eye data. We extracted the eye position at specific time-points during the occluded epoch (final, last-occluded, and mid-occlusion). For each of these timepoints, we defined an error metric computed as the difference between the vertical eye position and the vertical ball position. We then compared the pattern of errors derived from this new error metric to the pattern of (endpoint paddle) errors of all tested RNN, resulting in new consistency scores. Each row shows the results for one such metric, with these new RNN consistency scores plotted against (left) RNN simulation ability and (middle) RNN task performance. The right plot shows the proportion of variance in consistency scores explained by RNN simulation ability and RNN task performance. Over all metrics, we observe results which are largely consistent with the corresponding results using paddle position (Figure 3F-H).

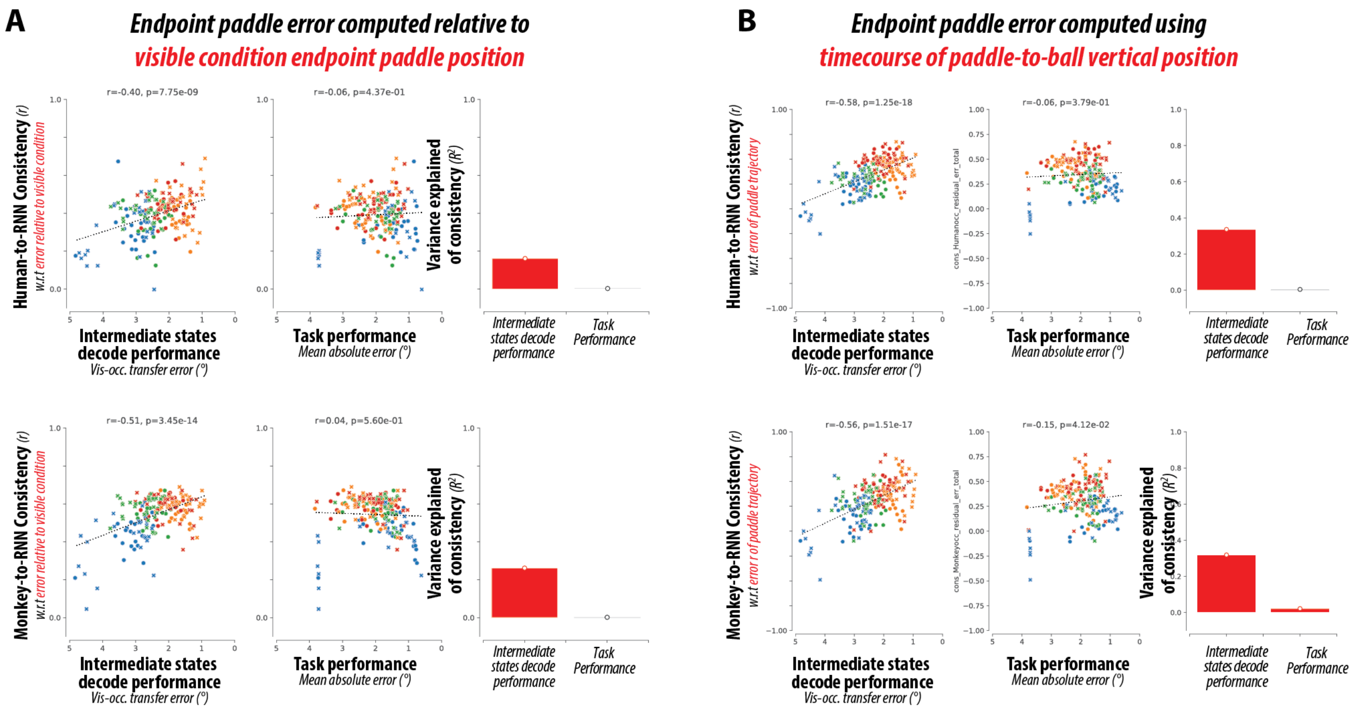

Figure S9. Alternative behavioral metrics. We repeated the primary analyses of Figure 3 (F, G,H) using alternative behavioral metrics. Formatting is identical to Figure S8C but includes both human and monkey data for measuring consistency scores.

(A) We defined an error vector that measures the paddle error as the difference between the endpoint paddle position on trials with a fully opaque occluder and the *endpoint paddle position on trials with a translucent occluder* ("visible" trials), rather than the ground truth endpoint ball position. The four panels, formatted as in Figure S8C, show the replication of the primary analyses using this newly defined behavioral error vector; we observe results consistent with Figure 3F-H.

(B) We defined an error vector that measures the paddle error as the difference between the *time course* of paddle position and ball position *during the entire occluded epoch*, rather than just the endpoint positions. The four panels, formatted as in Figure S8C, show the replication of the primary analyses using this newly defined behavioral error vector; we observe results consistent with Figure 3F-H.

Supplementary References

Andalman, Aaron S., Vanessa M. Burns, Matthew Lovett-Barron, Michael Broxton, Ben Poole, Samuel J. Yang, Logan Grosenick, et al. 2019. “Neuronal Dynamics Regulating Brain and Behavioral State Transitions.” *Cell* 177 (4): 970–85.e20.

Battaglia, Peter W., Jessica B. Hamrick, and Joshua B. Tenenbaum. 2013. “Simulation as an Engine of Physical Scene Understanding.” *Proceedings of the National Academy of Sciences* 110 (45): 18327–32.

Chaisangmongkon, Warasinee, Sruthi K. Swaminathan, David J. Freedman, and Xiao-Jing Wang. 2017. “Computing by Robust Transience: How the Fronto-Parietal Network Performs Sequential, Category-Based Decisions.” *Neuron* 93 (6): 1504–17.e4.

Collins, Jasmine, Jascha Sohl-Dickstein, and David Sussillo. 2016. “Capacity and Trainability in Recurrent Neural Networks.” *arXiv [stat.ML]*. arXiv. http://arxiv.org/abs/1611.09913.

Craik, K. J. W. 1952. *The Nature of Explanation*. CUP Archive.

DiCarlo, J. J., and K. O. Johnson. 1999. “Velocity Invariance of Receptive Field Structure in Somatosensory Cortical Area 3b of the Alert Monkey.” *The Journal of Neuroscience: The Official Journal of the Society for Neuroscience* 19 (1): 401–19.

Egger, Seth W., Evan D. Remington, Chia-Jung Chang, and Mehrdad Jazayeri. 2019. “Internal Models of Sensorimotor Integration Regulate Cortical Dynamics.” *Nature Neuroscience* 22 (11): 1871–82.

Fischer, Jason, John G. Mikhael, Joshua B. Tenenbaum, and Nancy Kanwisher. 2016. “Functional Neuroanatomy of Intuitive Physical Inference.” *Proceedings of the National Academy of Sciences* 113 (34): E5072–81.

Funahashi, Ken-Ichi, and Yuichi Nakamura. 1993. “Approximation of Dynamical Systems by Continuous Time Recurrent Neural Networks.” *Neural Networks*. https://doi.org/10.1016/s0893-6080(05)80125-x.

Gao, Peiran, Eric Trautmann, Byron M. Yu, Gopal Santhanam, Stephen Ryu, Krishna Shenoy, and Surya Ganguli. 2017. “A Theory of Multineuronal Dimensionality, Dynamics and Measurement.” *bioRxiv*. https://doi.org/10.1101/214262.

Gerstenberg, Tobias, and Joshua B. Tenenbaum. 2017. “Intuitive Theories.” *Oxford Handbook of Causal Reasoning*, 515–48.

Golub, Matthew, and David Sussillo. 2018. “FixedPointFinder: A Tensorflow Toolbox for Identifying and Characterizing Fixed Points in Recurrent Neural Networks.” *Journal of Open Source Software* 3 (31): 1003.

Goodfellow, Ian J., Jean Pouget-Abadie, Mehdi Mirza, Bing Xu, David Warde-Farley, Sherjil Ozair, Aaron Courville, and Yoshua Bengio. 2014. “Generative Adversarial Networks.” *arXiv Preprint arXiv:1406. 2661* 4 (5): 6.

Hammer, Barbara. 2000. “On the Approximation Capability of Recurrent Neural Networks.” *Neurocomputing* 31 (1): 107–23.

Hamrick, Jessica B. 2019. “Analogues of Mental Simulation and Imagination in Deep Learning.” *Current Opinion in Behavioral Sciences* 29 (October): 8–16.

Hassabis, Demis, Dharshan Kumaran, and Eleanor A. Maguire. 2007. “Using Imagination to Understand the Neural Basis of Episodic Memory.” *The Journal of Neuroscience: The Official Journal of the Society for Neuroscience* 27 (52): 14365–74.

Hegarty, Mary. 2004. “Mechanical Reasoning by Mental Simulation.” *Trends in Cognitive Sciences* 8 (6): 280–85.

Hessel, Matteo, Joseph Modayil, Hado van Hasselt, Tom Schaul, Georg Ostrovski, Will Dabney, Dan Horgan, Bilal Piot, Mohammad Azar, and David Silver. 2017. “Rainbow: Combining Improvements in Deep Reinforcement Learning.” *arXiv [cs.AI]*. arXiv. http://arxiv.org/abs/1710.02298.

Higgins, Irina, Loic Matthey, Arka Pal, Christopher Burgess, Xavier Glorot, Matthew Botvinick, Shakir Mohamed, and Alexander Lerchner. 2016. “Beta-VAE: Learning Basic Visual Concepts with a Constrained Variational Framework.” https://openreview.net/pdf?id=Sy2fzU9gl.

Hoch, Stephen J. 1985. “Counterfactual Reasoning and Accuracy in Predicting Personal Events.” *Journal of Experimental Psychology. Learning, Memory, and Cognition* 11 (4): 719–31.

Johnson, Kenneth O., Steven S. Hsiao, and Takashi Yoshioka. 2002. “Neural Coding and the Basic Law of Psychophysics.” *The Neuroscientist: A Review Journal Bringing Neurobiology, Neurology and Psychiatry* 8 (2): 111–21.

Kanitscheider, Ingmar, and Ila Fiete. 2017. “Training Recurrent Networks to Generate Hypotheses about How the Brain Solves Hard Navigation Problems.” In *Advances in Neural Information Processing Systems*, edited by I. Guyon, U. V. Luxburg, S. Bengio, H. Wallach, R. Fergus, S. Vishwanathan, and R. Garnett, 30:4529–38. Curran Associates, Inc.

Kell, A. J. E., Daniel L. K. Yamins, Erica N. Shook, Sam V. Norman-Haignere, and Josh H. McDermott. 2018. “A Task-Optimized Neural Network Replicates Human Auditory Behavior, Predicts Brain Responses, and Reveals a Cortical Processing Hierarchy.” *Neuron* 98 (3): 630–44.e16.

Kulkarni, Tejas D., William F. Whitney, Pushmeet Kohli, and Josh Tenenbaum. 2015. “Deep Convolutional Inverse Graphics Network.” In *Advances in Neural Information Processing Systems*, edited by C. Cortes, N. Lawrence, D. Lee, M. Sugiyama, and R. Garnett, 28:2539–47. Curran Associates, Inc.
Ladenbauer, Josef, Sam McKenzie, Daniel Fine English, Olivier Hagens, and Srdjan Ostojic. 2019. “Inferring and Validating Mechanistic Models of Neural Microcircuits Based on Spike-Train Data.” *Nature Communications* 10 (1): 4933.
LeCun, Yann, Yoshua Bengio, and Geoffrey Hinton. 2015. “Deep Learning.” *Nature* 521 (7553): 436–44.
Lee, Hyodong, and James J. DiCarlo. 2019. “Topographic Deep Artificial Neural Networks (TDANNs) Predict Face Selectivity Topography in Primate Inferior Temporal (IT) Cortex.” *arXiv [q-bio.NC]*. arXiv. http://arxiv.org/abs/1909.09847.
Lerer, Adam, Sam Gross, and Rob Fergus. 2016. “Learning Physical Intuition of Block Towers by Example.” *arXiv [cs.AI]*. arXiv. http://arxiv.org/abs/1603.01312.
Maheswaranathan, N., A. Williams, and M. Golub. 2019. “Universality and Individuality in Neural Dynamics across Large Populations of Recurrent Networks.” *Advances in Neural Information Processing Systems*. http://papers.nips.cc/paper/9694-universality-and-individuality-in-neural-dynamics-across-large-populations-of-recurrent-networks.
Mante, Valerio, David Sussillo, Krishna V. Shenoy, and William T. Newsome. 2013. “Context-Dependent Computation by Recurrent Dynamics in Prefrontal Cortex.” *Nature* 503 (7474): 78–84.
Marblestone, Adam H., Greg Wayne, and Konrad P. Kording. 2016. “Toward an Integration of Deep Learning and Neuroscience.” *Frontiers in Computational Neuroscience* 10 (September): 94.
Mastrogiuseppe, Francesca, and Srdjan Ostojic. 2018. “Linking Connectivity, Dynamics, and Computations in Low-Rank Recurrent Neural Networks.” *Neuron* 99 (3): 609–23.e29.
Michaels, Jonathan A., Benjamin Dann, and Hansjörg Scherberger. 2016. “Neural Population Dynamics during Reaching Are Better Explained by a Dynamical System than Representational Tuning.” *PLoS Computational Biology* 12 (11): e1005175.
Michaels, Jonathan A., Stefan Schaffelhofer, Andres Agudelo-Toro, and Hansjörg Scherberger. 2019. “A Neural Network Model of Flexible Grasp Movement Generation.” https://doi.org/10.1101/742189.
Nalisnick, Eric, Akihiro Matsukawa, Yee Whye Teh, Dilan Gorur, and Balaji Lakshminarayanan. 2018. “Do Deep Generative Models Know What They Don’t Know?” *arXiv [stat.ML]*. arXiv. http://arxiv.org/abs/1810.09136.
Nishimoto, Shinji, and Jack L. Gallant. 2011. “A Three-Dimensional Spatiotemporal Receptive Field Model Explains Responses of Area MT Neurons to Naturalistic Movies.” *The Journal of Neuroscience: The Official Journal of the Society for Neuroscience* 31 (41): 14551–64.
Remington, Evan D., Devika Narain, Eghbal A. Hosseini, and Mehrdad Jazayeri. 2018. “Flexible Sensorimotor Computations through Rapid Reconfiguration of Cortical Dynamics.” *Neuron* 98 (5): 1005–19.e5.
Richards, Blake A., Timothy P. Lillicrap, Philippe Beaudoin, Yoshua Bengio, Rafal Bogacz, Amelia Christensen, Claudia Clopath, et al. 2019. “A Deep Learning Framework for Neuroscience.” *Nature Neuroscience* 22 (11): 1761–70.
Russo, Abigail A., Sean R. Bittner, Sean M. Perkins, Jeffrey S. Seely, Brian M. London, Antonio H. Lara, Andrew Miri, et al. 2018. “Motor Cortex Embeds Muscle-like Commands in an Untangled Population Response.” *Neuron* 97 (4): 953–66.e8.
Sarafyazd, Morteza, and Mehrdad Jazayeri. 2019. “Hierarchical Reasoning by Neural Circuits in the Frontal Cortex.” *Science* 364 (6441). https://doi.org/10.1126/science.aav8911.
Schrimpf, Martin, Jonas Kubilius, Ha Hong, Najib J. Majaj, Rishi Rajalingham, Elias B. Issa, Kohitij Kar, et al. 2018. “Brain-Score: Which Artificial Neural Network for Object Recognition Is Most Brain-Like?” *bioRxiv*. https://doi.org/10.1101/407007.
Shepard, R. N., and J. Metzler. 1971. “Mental Rotation of Three-Dimensional Objects.” *Science* 171 (3972): 701–3.
Sohn, Hansem, Devika Narain, Nicolas Meirhaeghe, and Mehrdad Jazayeri. 2019. “Bayesian Computation through Cortical Latent Dynamics.” *Neuron* 103 (5): 934–47.e5.
Spearman, Charles. 1961. “The Proof and Measurement of Association between Two Things.” https://psycnet.apa.org/record/2006-10257-005.
Storrs, Katherine R., and Nikolaus Kriegeskorte. 2019. “Deep Learning for Cognitive Neuroscience.” *arXiv [q-bio.NC]*. arXiv. http://arxiv.org/abs/1903.01458.
Sussillo, David, and Omri Barak. 2013. “Opening the Black Box: Low-Dimensional Dynamics in High-Dimensional Recurrent Neural Networks.” *Neural Computation* 25 (3): 626–49.
Sussillo, David, Mark M. Churchland, Matthew T. Kaufman, and Krishna V. Shenoy. 2015. “A Neural Network That Finds a Naturalistic Solution for the Production of Muscle Activity.” *Nature Neuroscience* 18 (7): 1025–33.
Tenenbaum, Joshua B., Charles Kemp, Thomas L. Griffiths, and Noah D. Goodman. 2011. “How to Grow a Mind: Statistics, Structure, and Abstraction.” *Science* 331 (6022): 1279–85.

Ullman, Tomer D., Elizabeth Spelke, Peter Battaglia, and Joshua B. Tenenbaum. 2017. “Mind Games: Game Engines as an Architecture for Intuitive Physics.” *Trends in Cognitive Sciences* 21 (9): 649–65.
Wang, Jing, Devika Narain, Eghbal A. Hosseini, and Mehrdad Jazayeri. 2018. “Flexible Timing by Temporal Scaling of Cortical Responses.” *Nature Neuroscience* 21 (1): 102–10.
Yamins, Daniel L. K., Ha Hong, Charles F. Cadieu, Ethan A. Solomon, Darren Seibert, and James J. DiCarlo. 2014. “Performance-Optimized Hierarchical Models Predict Neural Responses in Higher Visual Cortex.” *Proceedings of the National Academy of Sciences of the United States of America* 111 (23): 8619–24.
Yang, Guangyu Robert, Michael W. Cole, and Kanaka Rajan. 2019. “How to Study the Neural Mechanisms of Multiple Tasks.” *Current Opinion in Behavioral Sciences* 29 (October): 134–43.
Yang, Guangyu Robert, Madhura R. Joglekar, H. Francis Song, William T. Newsome, and Xiao-Jing Wang. 2019. “Task Representations in Neural Networks Trained to Perform Many Cognitive Tasks.” *Nature Neuroscience* 22 (2): 297–306.
Zacks, Jeffrey M. 2008. “Neuroimaging Studies of Mental Rotation: A Meta-Analysis and Review.” *Journal of Cognitive Neuroscience* 20 (1): 1–19.
