# Supplementary material for: Recurrent neural networks with explicit representation of dynamic latent variables can mimic behavioral patterns in a physical inference task
Source: Nat Commun. 2022 Oct 4;13:5865. doi: 10.1038/s41467-022-33581-6 (PMC9532407; doi:10.1038/s41467-022-33581-6)
Supplement: Supplementary file 3 — Reporting Summary [file 41467_2022_33581_MOESM3_ESM.pdf]

nature portfolio

Corresponding author(s): Jazayeri

Last updated by author(s): 08/30/2022

Reporting Summary

Nature Portfolio wishes to improve the reproducibility of the work that we publish. This form provides structure for consistency and transparency in reporting. For further information on Nature Portfolio policies, see our Editorial Policies and the Editorial Policy Checklist.

Statistics

For all statistical analyses, confirm that the following items are present in the figure legend, table legend, main text, or Methods section.

n/a	Confirmed	
[ ]	[x]	The exact sample size (*n*) for each experimental group/condition, given as a discrete number and unit of measurement
[ ]	[x]	A statement on whether measurements were taken from distinct samples or whether the same sample was measured repeatedly
[ ]	[x]	The statistical test(s) used AND whether they are one- or two-sided<br>*Only common tests should be described solely by name; describe more complex techniques in the Methods section.*
[ ]	[x]	A description of all covariates tested
[ ]	[x]	A description of any assumptions or corrections, such as tests of normality and adjustment for multiple comparisons
[ ]	[x]	A full description of the statistical parameters including central tendency (e.g. means) or other basic estimates (e.g. regression coefficient) AND variation (e.g. standard deviation) or associated estimates of uncertainty (e.g. confidence intervals)
[ ]	[x]	For null hypothesis testing, the test statistic (e.g. *F*, *t*, *r*) with confidence intervals, effect sizes, degrees of freedom and *P* value noted<br>*Give P values as exact values whenever suitable.*
[x]	[ ]	For Bayesian analysis, information on the choice of priors and Markov chain Monte Carlo settings
[x]	[ ]	For hierarchical and complex designs, identification of the appropriate level for tests and full reporting of outcomes
[ ]	[x]	Estimates of effect sizes (e.g. Cohen's *d*, Pearson's *r*), indicating how they were calculated

Our web collection on statistics for biologists contains articles on many of the points above.

Software and code

Policy information about availability of computer code

Data collection	Behavioral data was collected using MWorks (mworks.github.io, version 0.9). RNNs were trained using tensorflow 1.14.
Data analysis	Data analysis was performed with python code, using python libraries including numpy, scipy, scikit-learn, pandas, matplotlib, and seaborn. Version numbers for all libraries and requirements are listed on https://github.com/RishiRajalingham/MPongBehavior_public.

For manuscripts utilizing custom algorithms or software that are central to the research but not yet described in published literature, software must be made available to editors and reviewers. We strongly encourage code deposition in a community repository (e.g. GitHub). See the Nature Portfolio guidelines for submitting code & software for further information.

Data

Policy information about availability of data

All manuscripts must include a data availability statement. This statement should provide the following information, where applicable:

- Accession codes, unique identifiers, or web links for publicly available datasets
- A description of any restrictions on data availability
- For clinical datasets or third party data, please ensure that the statement adheres to our policy

The pre-processed data used to generate the associated figures are available on a public repository (https://github.com/RishiRajalingham/MPongBehavior_public). All main and supplemental figures can be reproduced using the notebooks and raw data stored in this repository. Code for reproducing raw data for RNNs is made available at the same repository. Raw data for human and monkey behavior is not shared due to dataset size and complexity, but will be made available upon request to the corresponding author.

Human research participants

Policy information about studies involving human research participants and Sex and Gender in Research.

Reporting on sex and gender	We did not collect gender/sex information of human participants.
Population characteristics	We did not collect covariate information of human participants.
Recruitment	Participants were recruited by an email list.
Ethics oversight	Committee on the Use of Humans as Experimental Subjects at Massachusetts Institute of Technology

Note that full information on the approval of the study protocol must also be provided in the manuscript.

Field-specific reporting

Please select the one below that is the best fit for your research. If you are not sure, read the appropriate sections before making your selection.

☒ Life sciences ☐ Behavioural & social sciences ☐ Ecological, evolutionary & environmental sciences

For a reference copy of the document with all sections, see nature.com/documents/nr-reporting-summary-flat.pdf

Life sciences study design

All studies must disclose on these points even when the disclosure is negative.

Sample size	We used n=2 monkeys, which is standard practice for primate neuroscience. We used n=12 human subjects, which is within the range of standard practice for human neuroscience.
Data exclusions	We did not exclude any data relevant to this study.
Replication	All results reported were replicated by cross-validation across different train and test splits of the data, using k=2 splits of cross-validation.
Randomization	M-Pong conditions were presented to animals/humans in a randomized order. Moreover, all our statistics and results were cross validated by randomly subsampling a part of the data collected, and re-doing the analyses on that specific part.
Blinding	The presentation of the M-Pong conditions were randomly interleaved and therefore both the experimenters and the subjects were blind to the experimental conditions/groupings.

Reporting for specific materials, systems and methods

We require information from authors about some types of materials, experimental systems and methods used in many studies. Here, indicate whether each material, system or method listed is relevant to your study. If you are not sure if a list item applies to your research, read the appropriate section before selecting a response.

Materials & experimental systems

n/a	Involved in the study
[x]	[ ] Antibodies
[x]	[ ] Eukaryotic cell lines
[x]	[ ] Palaeontology and archaeology
[ ]	[x] Animals and other organisms
[x]	[ ] Clinical data
[x]	[ ] Dual use research of concern

Methods

n/a	Involved in the study
[x]	[ ] ChIP-seq
[x]	[ ] Flow cytometry
[x]	[ ] MRI-based neuroimaging

Animals and other research organisms

Policy information about studies involving animals; ARRIVE guidelines recommended for reporting animal research, and Sex and Gender in Research

Laboratory animals	2 adult female rhesus macaque monkeys (Macaca mulatta)
Wild animals	Study did not involve wild animals.
Reporting on sex	Study included both sexes. The effect of sex was not investigated.
Field-collected samples	Study did not involve field-collected samples.
Ethics oversight	All procedures were performed in compliance with the guideline of National Institutes of Health and the American Physiological Society, and approved by the MIT Committee on Animal Care.

Note that full information on the approval of the study protocol must also be provided in the manuscript.
